# A Simple Method for the Detection of Biofilms Using a Heatable Capacitive Sensor Structure (CSS): Description, Proof of Concept, and Some Technical Improvements

**DOI:** 10.3390/s22020445

**Published:** 2022-01-07

**Authors:** Kai-Uwe Zirk, Manuel Olze, Harald Pötzschke

**Affiliations:** ZME–Centre for Mechatronics and Electronics, PHWT–Private University for Economics and Engineering, Thüringer Str. 3A, D-49356 Diepholz, Germany; manuel.olze@gmx.de (M.O.); poetzschke@web.de (H.P.)

**Keywords:** microbial, biofilm, capacitance, capacitive sensor structure, heating sensor, hydrogel, PCB, bacteria

## Abstract

This article presents a novel method for the detection of biofilms based on a heatable, capacitive sensor structure (CSS). Biofilms are capable of strongly binding large amounts of water to their extracellular biopolymer matrix, which is detectable via its dielectric properties. A main challenge is to determine the difference between the inherent occurring presence of moisture in the ecosystem, which is necessary to form a biofilm and an actual formed biofilm. Therefore, the CSS is carefully heated to evaporate unbound surface moisture and determine whether there is a remaining residual alternation of the capacitance in comparison to the dry state. As a reproduceable substitute for complex, real biofilms, a hygroscopic, medical hydrogel-based on polysaccharides was used and applied by spray coating. Printed circuit boards (PCB) in different geometries and materials were used as CSS and compared in terms of their performance. A layer-thickness of 20 µm for the hydrogel coating to be sufficiently detected was defined as a realistic condition based on known values for real biofilms cited in literature. For this thickness a double-meander structure proves to be preferable over interdigitating and spiral geometries. It does offer a 30% lower, yet sufficient sensitivity, but shows advantages in manufacturing (one layer instead of two) and conductive heating capability. In the experiments, free water showed virtually no residual change, while the hydrogel-coated CSS still shows an approx. 300% higher value compared to a dry capacity. Yet, the overall small capacities of about 6–30 pF in dry state are difficult to measure and therefore sensitive to interferences and noise, which results in a high deviation. The principle of measurement can be evaluated as proofed by the carried out experiments, though offering room for improvement in the design of the study. The new method might be especially useful for pipes (e.g., hydrodynamically ineffective sensors installed in a pipe wall) if they at least are not permanently flooded with an aqueous medium, but can occasionally dry. If the internal surface is still only moist, it can be dried by initial heating.

## 1. Introduction

The vast majority of microbial life is believed to exist in biofilms [1]. Biofilms are slimy layers formed by microorganisms that are themselves embedded in them. In many applications, the possible consequences of the formation of biofilms are undesirable [2]. Therefore, several methods to detect biofilms have been developed in recent decades [3,4,5,6,7,8]. Each method has to meet various requirements depending on what it is used for. While highly sensitive techniques involving sampling and laboratory testing are often used in medicine [7,8], more robust methods for long-term monitoring are needed in infrastructure systems. Biofilms occur in a wide variety of different habitats [1]. Because their composition varies so much, it is hard to generalize in detail about their properties such as heterogeneity, bacterial species diversity, and thickness [9] (the realistic modelling of biofilms in their complexity is a separate area of research [10,11,12]). Even so, some basic aspects can be summarized as follows. The formation of a biofilm is initially preceded by bacteria sticking to a surface [1]. Depending on the specific bacterial strain, a bacterial culture is formed, and an extracellular polymeric matrix is produced [13]. Mainly consisting of polysaccharides [9,14], this matrix binds large amounts of water, accounting for up to 97% of the biofilm [15]. The bacteria and the extracellular polymer matrix act together to form the biofilm. Over time, it also offers other bacterial species a favourable habitat, so monocultural biofilms (biofilms with just one bacterial species) are rare under natural conditions [9]. To sum up, certain conditions must be met for the formation of a biofilm, namely:

1. The bacteria must be able to stick to the specific surface and produce an extracellular polymer matrix.
2. The conditions must be suitable for bacteria to thrive. For example, there must be an adequate supply of oxygen and nutrients, especially carbon compounds.
3. The ambient moisture must be sufficiently high as a result of humidity and/or condensation, regular wetting or flooding, or the ecosystem being permanently underwater.

The detection of biofilms on the basis of their dielectric or thermal properties is an established approach [4,5,6,7,16]. Frequently, electrical frequency spectroscopy is used, which repays its comparatively complex setup with high specificity when it comes to distinguishing bacterial biofilms from other dielectrically active substances [5].

A much simpler method of detecting a biofilm could be to capture the biofilm’s dielectric properties (relative permittivity, i.e., the permeability of a material to electric fields) if there is no accompanying free water (i.e., the electrical capacitance of the sensor structure is only changed by the matrix-bound water in the biofilm). Water—including when bound to the matrix of a biofilm—has a relative permittivity about 80 times higher than air. Sensors with a hygroscopic surface, for example, which are used to detect air humidity, also work by capturing dielectric properties [17,18,19]. However, one snag when it comes to detecting a biofilm via its moisture content is the simultaneous presence of ambient moisture, which is always a basic requirement for the formation of a biofilm (see above). The difference is that, unlike ambient moisture, biofilms bind water in the biofilm matrix [9,15]. Therefore, a biofilm dries only very slowly and—unlike (unbound, freely evaporable) surface water—the matrix-bound water forms a dielectrically effective “residue” over a certain period of time.

For proof of concept and an initial assessment of the most suitable boundary conditions of the new, theoretically derived method to detect biofilms, both flexible (polyimide) and rigid (ceramic) sensor structures along with a control unit for electrical heating and measurement were produced and tested for their effectiveness. Despite their considerable functional disadvantages (see below), flexible sensor structures were mainly used to determine the most suitable geometrical structure because they were simpler and much cheaper to make. This geometry was then also produced as a rigid ceramic structure, a more elaborate process. The new concept involved heating the sensor structures until the free water had been completely evaporated. The changes in electrical capacitance were measured in the presence and absence of free water or (in the case of rigid ceramic structures) of a technical hydrogel as a biofilm model.

**Figure 1:**
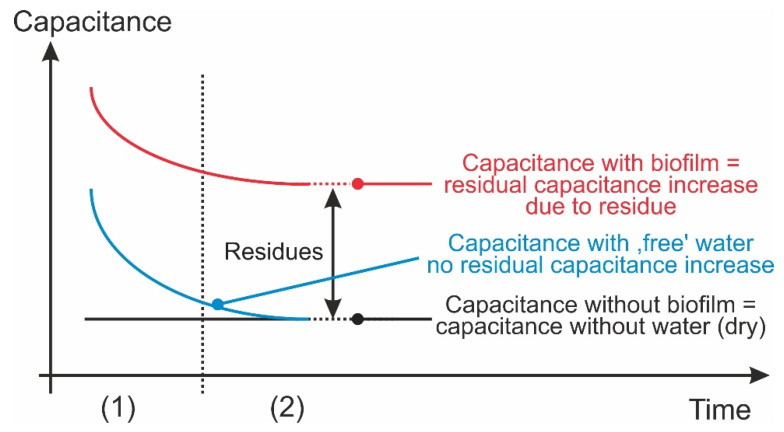
Functional principle of the process, showing the theoretical (idealized) time courses of capacitance due to heating, which are not affected by the actual temperatures. The procedure consists of (1) a (standardized) heating phase, in which water on the surface of the structure not bound to the biofilm matrix evaporates completely, while matrix-bound water remains predominantly (or at least partially) on the surface, and (2) a cooling phase, in which undisturbed ambient conditions are re-established. During both phases, the electrical capacitance of the sensor structure is measured.

According to the new method, a biofilm is detected by determining the relative permittivity of residual, matrix-bound water on the surface above the sensor structure (indirectly by measuring the electrical capacitance) once the water not bound to a matrix (i.e., the “free” water only wetting the surface) has been completely removed by the standardized heating of the surface. Comparison with values without any water on the surface reveals the change in capacitance due to the matrix-bound water (see the diagram in Figure 1). The following boundary conditions apply to the detection of biofilm formation in technical systems:

1. The sensors must not hydrodynamically obstruct flowing liquids. They should therefore be seamlessly integrated into smooth surfaces.
2. To allow free surface water to be removed, the installation point must not be constantly underwater. Instead, the majority of the free water must be able to drain off freely before measurement takes place.

## 2. Materials and Methods

### 2.1. Sensor Structures

#### 2.1.1. Modulated Sensor Structures

In the first step, suitable-seeming flat (planar) electrically conductive sensor structures were designed and their electrical behaviour was initially modulated (PC-based).

#### 2.1.2. Flexible Sensor Structures

**Figure 2:**
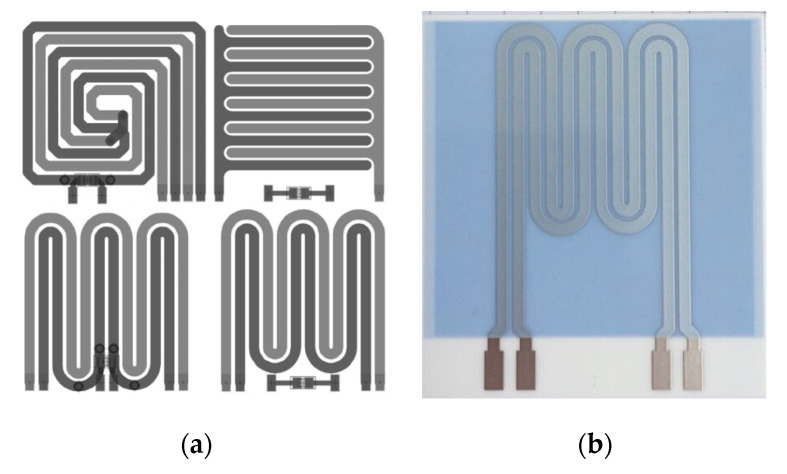
Schematic pictures of the produced sensor structures. (**a**) Sensor structures produced as flexible PCBs (top left to bottom right): double spiral structure S1, comb structure K1, double meander structures M1 and M2. Although the two electrical conductors (electrodes) are both made of (identical) copper, they are shown in different colours for reasons of clarity. (**b**) Photo of a rigid PCB with double meander structure M2 (ceramic substrate material).

Two companies (LeitOn, Berlin, Germany and BetaLAYOUT, Aarbergen, Germany) were commissioned to produce the modulated sensor structures with suitable-seeming geometries as standard flexible polyimide printed circuit boards (PCBs). The PCBs measured approximately 32 mm × 32 mm, and the entire surface was filled with the sensor structures. Diagrams of the structures are shown on the left in Figure 2 and described in Table 1. According to one manufacturer polyimide (the substrate material) has a moisture absorption rate of 3% [20], which must be taken into account when the PCB is wetted with water.

#### 2.1.3. Rigid Sensor Structure

Turck Duotec, Halver, was commissioned to produce the sensor structure double meander M2 as a rigid PCB with identical geometry to the flexible polyimide PCB apart from the different thickness of the conductive layer (see Figure 2b).

The sensor structure double meander M2 is universally suitable. For one thing, it doesn’t require any electrical through-plating of the PCB for the return of the conductor layer like the sensor structure double spiral S1 (see Figure 2a). Moreover, by using the double meander structure M2, both the heating and the measurement of the electrical capacitance can be carried out with the same sensor conductor layer, unlike for example the comb structure K1. The substrate material of the rigid PCBs (length × width × height = 50.8 mm × 46 mm × 1 mm) was an aluminium oxide ceramic (‘CoorsTek ADS-96R’) to which silver conductor conductors (height = 10 µm) were initially applied by sintering, which were then sealed with a glass layer (thickness = 22 µm) to reliably prevent the absorption of moisture (like the substrate material polyimide of the flexible conductors).

### 2.2. Heating Process

The flexible PCBs were heated using a separate heating foil ‘THF-4760’ 12 V, 3 W (thermo Flächenheizungssysteme, Rohrbach, Germany) fixed directly beneath the PCB. For the rigid PCBs, heating was initially carried out using a ‘C-MAG HS 7’ controlled heating plate (IKA, Staufen, Germany) placed underneath to enable uniform, homogeneous heating. However, since an essential part of the concept for the detection of biofilms is the controlled heating of the sensor structure in order to remove unbound water by means of evaporation, subsequently, the sensor structure itself was used for heating. If the conductors are used as heating resistors, additional heating resistors aren’t required. In addition, using the conductors as heating resistors ensures heat input close to the surface, enabling quicker response times. For heating purposes, the galvanically separated conductors of the rigid PCB were electrically connected using an electromechanical relay to achieve relatively high ohmic (thermal) resistance. Throughout the heating process, after every 15 s this resistance was interrupted for one second, during which capacitance was measured. An “8722-2” external laboratory power supply unit (Toellner, Herdecke, Germany) with a maximum electrical power rating of approximately 72 W was always used as the power source for the heating current.

### 2.3. Capacitance Measurement

Electrical capacitance was measured with either (1) an LCR meter ‘HM8118’ (Rohde & Schwarz, Munich, Germany), (2) a ‘Precision Impedance Analyzer WK 6500/6505B’ (Wayne Kerr Electronics, Chichester, UK), and/or (3) an internally designed and built measuring apparatus (described in detail in Section 2.5).

### 2.4. Temperature Measurement

Keevil et al. showed in experiments on hot water circuits that biofilm growth occurs up to a temperature of approximately 55 °C [21]. Therefore, in order to avoid temperatures that could potentially kill bacteria and change the biofilm before it is detected, the surface temperature of the flexible PCB was measured with a platinum measuring resistor (Pt1000), and also thermographically using a thermal imaging camera ‘Testo 885’ (Testo, Titisee-Neustadt, Germany). However, differences of up to 20 °C were recorded between the Pt1000 and the polyimide PCB surface, presumably due to poor thermal coupling. Accordingly, for the rigid PCBs, the temperature was not simultaneously measured with a platinum measuring resistor. A maximum temperature of 60 °C reached at the end of a maximum heating period of 10 min was chosen as the critical temperature.

### 2.5. Internally Built Measuring Apparatus

**Figure A1:**
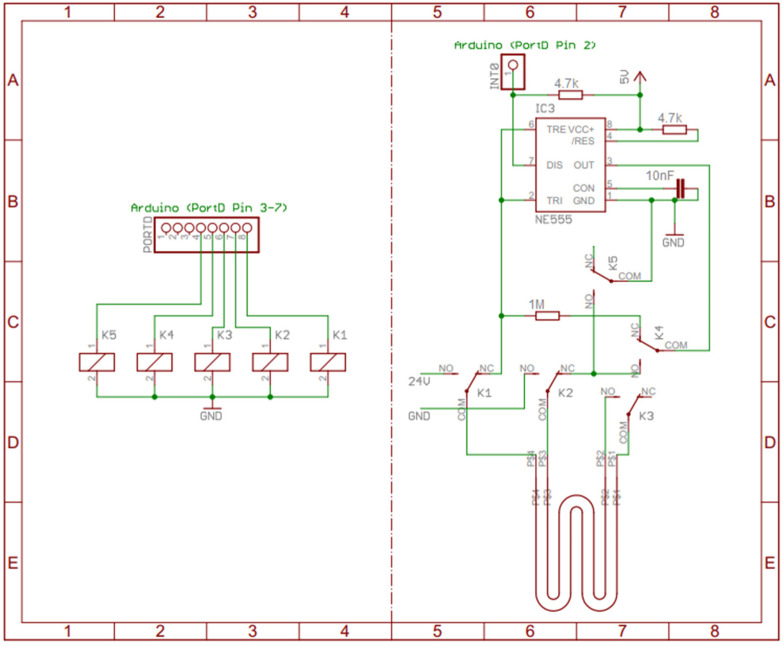
Schematic of the used time-to-digital-conversion-circuit for capacitance-measurement based on an Arduino Uno described in Section 2.5. Two Modes of Operation are to be differed: (1) Heating the CSS, for higher ohmic resistance both tracks are switched in row—K1/K2/K3 switched to NO, K4/K5 switched to NC. (2) Measuring the capacitance of the CSS by charging it with a 1 MOhm Resistor—K1/K2/K3 switched to NC, K4/K5 switched to NO.

The internally made apparatus was automatically controlled using an Arduino Uno microcontroller. Its main goal was the automation of the system to ease the whole research at this early state of investigation (Proof of Principle). Hence the amount of improving it for highest resolution and accuracy was held low and it was constantly compared to the calibrated laboratory instruments. To fulfil the previously described tasks of (1) heat the CSS, (2) measure the capacitance and (3) log the data, additional circuitry was necessary. For datalogging it was supplemented with a commercial SD card shield. To switch between the different states of heating and capacitance measurement electromechanical relais were used (See Appendix A, Figure A1). Brief experiments with MOSFETs and solid-state relais were cancelled in early states since their parasitic capacitance heavily affected the measurement. For heating, an external power supply of 24 V and a maximum current of 5 A was given. The electrical capacitance was determined indirectly using a simple time-to-digital method. In this process, the (capacitive) structure (C) is electrically connected to an RC element via a fixed, known ohmic resistance (R). In this setup each of the tracks represents one electrode of a plane capacitor. An NE555-IC was used as an a-stable oscillator-circuit. In this configuration the NE555 does charge and discharge the connected RC element alternating between an upper threshold-voltage of 2/3 of its own supply (Vcc) and a lower threshold of 1/3 Vcc by generating a square-wave-signal at its own output. As the resistor has a fixed value, the change of capacitance of the CSS in wetted state directly alters the switching frequency of the NE555. In result the timing between two falling edges on its output is elongated, when the CSS is wet. Catching two edges as interrupt-requests with the microcontroller and use the internal quarz based frequency of 16 MHz as a stopwatch makes it possible to measure the capacitance, if the Resistor has a fixed, known value. The Datasheet of the NE555 names a minimum stable operating period of 10 µs for a full switching cycle [22]. This does limit the detectable minimal capacities to 7.2 pF according to the formula given in the datasheet for the known values (see Equations (1) and (2)). Though, as the NE555 is an analog IC, the minimum period of 10 µs is not to be understood as an increment for the resolution at the same time. The scanning frequency of the microcontroller with 16 MHz (62.5 ns period time) is resulting in a resolution of about 45 fF, in theory (see Equation (3)).

(1) $$C=\frac{T}{2\times 0.693\times R}$$

(2) $$C=\frac{10\times 10^{-6}s}{2\times 0.693\times 10^{6}\Omega }=7.2\times 10^{-12}F$$

(3) $$C=\frac{62.5\times 10^{-9}s}{2\times 0.693\times 10^{6}\Omega }=45.1\times 10^{-15}F$$

If the element is controlled with a square-wave signal, the frequency of the signal changes depending on the structure’s capacitance. To calibrate the correlation between frequency and capacitance, various capacitors with the electronic circuit of the measuring apparatus whose electrical capacitances had previously been measured with an LCR meter were fitted.

### 2.6. Hydrogel as a Biofilm Model

Real biofilms are subject to constant change. Since reproducible conditions are desirable for process validation, a technical biofilm model was initially chosen that imitated the properties of a real biofilm. The extracellular polymer matrix accounts for 50–90% of the organic carbon compounds within a biofilm and mainly consists of polysaccharides, as cited in [14]. Zhang et al. showed that biofilms have a water content of up to 97% [15]. Therefore, it was assumed that the dielectric and thermodynamic properties of a polysaccharide-based hydrogel closely resemble those of a real biofilm.

The thickness of real biofilms varies greatly depending on the bacteria involved and external influences. Stoodley et al. measured biofilm thicknesses of up to 130 μm [23]. James et al. describe biofilms of *Klebsiella pneumoniae* and *Pseudomonas aeruginosa* with thicknesses of 15–40 μm [24].

The medical hydrogel Vetericyn PLUS (Vetericyn, Rialto, CA, USA), which has low viscosity, was chosen because, like water, it could be sprayed onto the sensor structures in thin layers, enabling small amounts of wetting. Since this hydrogel consists of 99.97% water, its density can be assumed to be practically the same as that of water. For guidance, an average layer thickness per spray was determined experimentally from the hydrogel’s density, its mass (measured using a precision balance “Secura 225D-1s”, Sartorius, Göttingen), and the area covered (precision calliper gauge) (Table 2):

### 2.7. Proof of Concept: Experiments

**Figure 3:**
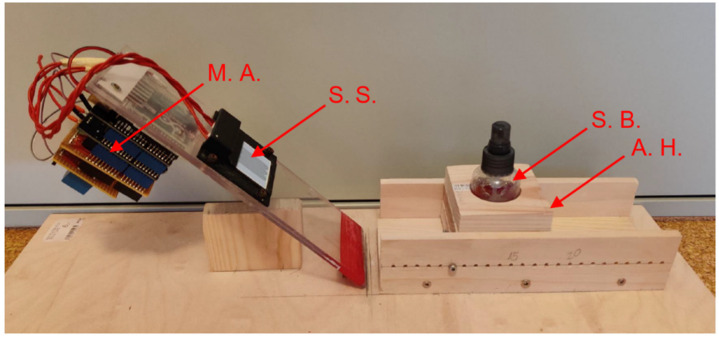
Photo of an experimental setup. Left: Sensor structure (S. S.) inclined by about 45° with measuring apparatus (M. A.) mounted on the back. Right: Adjustable holder (A. H.) for spray bottles (S. B.) used to spray water or hydrogel onto the sensor surface.

The sensor structures were mounted on an inclined plane at an angle of approximately 40–45° (to allow excess free water to run off easily during the wetting experiments). The measuring apparatus was firmly screwed to the back of the (flexible or rigid) PCB (see Figure 3). The measurements recorded using the measuring apparatus were verified using an “HM8118” calibrated LCR meter or a “WK 6500/6505B Precision Impedance Analyzer”.

#### 2.7.1. Flexible Sensor Structures (Polyimide PCBs)

The dry PCBs were slowly heated using a heating foil from approximately 20 °C to 60 °C, and the electrical capacitances of the sensor structures were measured before and after heating (HM8118, internally built measuring apparatus) to determine the temperature dependence of the PCB.

The PCBs were wetted with water from a commercially available spray bottle from a distance of about 5 cm at room temperature without being heated. Two spray bursts with a total volume of about 1 mL were applied. The electrical capacitances of the sensor structures were measured dry and with (free) water (HM8118 or internally made measuring apparatus). This enabled the highest (relative) differences in capacitance between dry and moist sensor structures to be determined.

The PCBs were sprayed with approximately 1 mL water from a distance of about 5 cm using a commercially available spray bottle as described and then heated with a heating foil from approximately 20 °C to 50 °C within 10 min. The electrical capacitances of the sensor structures as well as the temperatures determined with a Pt1000 resistor were continuously recorded (1 value per second) using the measuring apparatus. This allowed the time required to remove (free) surface water at a given (evaporation) temperature to be determined.

#### 2.7.2. Rigid Sensor Structures (Ceramic PCBs)

A dry PCB with a double meander structure was slowly heated from approximately 20 °C to 60 °C with a controlled heating plate. During this process, the capacitance of the structure was measured quasi-continuously (WK 6500/6505B) to determine the temperature dependence of the PCB.

The electrical capacitance of the PCBs’ structure was initially measured at room temperature (WK 6500/6505B). A PCB was then immersed in a beaker filled with distilled water at room temperature (to prevent an electrical short circuit without its electrical contacts) and the measurements were repeated. This enabled the maximum difference in capacitance between the dry sensor structure and the sensor structure completely wetted with (free) surface water to be determined.

To determine the thickness and homogeneity of the hydrogel sprayed on, an inclined PCB matching the size of the ceramic sensor structure was wetted from various distances (5 cm, 10 cm, 15 cm and 20 cm) by spraying on hydrogel different numbers of times without heating. This minimized effects due to evaporation (especially drying out), which might have falsified the results, especially with very thin layers of hydrogel.

Between being wetted again with hydrogel, the surfaces were immersed in a water bath for about 5 s and then in a beaker of isopropanol for approximately 5 s, and the surfaces were wiped carefully with a paper towel to shorten the evaporation time of the residual isopropanol. Before each use, the nozzle of the spray bottle containing hydrogel was rinsed with two (unused) spray bursts and wiped clean with a lint-free paper towel (optical lens paper). This spraying and wiping removed any air in the suction tube as well as any (dried) residue on the spray nozzle in order to maintain the same conditions every time hydrogel was sprayed on.

To determine hydrogel residue (i.e., as the biofilm model), the PCB was heated permanently (without a cooling phase) using its conductors. The flow of electric current of about 1.5 A (approx. 36 W at 24 V power supply) was only interrupted to measure the capacitance at each particular time using the measuring apparatus (Δt_heating_/Δt_measuring_ = 15). By omitting a cooling phase, time-dependent influences were minimized, and the final values of the capacitance (due to “residues”—residual capacitances) were reached more quickly during the heating phase. The duration of the heating phase was also limited so that “residues” were not changed greatly by the heating effect (in particular, so that thin layers did not dry out completely), which might have led to the biofilm model selected not working. At a spray distance of 20 cm, first only water and then hydrogel or different combinations of water and hydrogel sprays were applied to the surface. Between each measurement, both the PCBs and the spray nozzle were cleaned as described above.

## 3. Results

### 3.1. Flexible Sensor Structures (Polyimide PCBs)

Heating the PCBs with the heating foil from approximately 20 °C to 60 °C caused a relative increase in electrical capacitance of up to 50%.

The double spiral S1 and double meander M2 sensor structures, both with a copper layer thickness of 28 μm, were found to have a higher absolute capacitance both before and after spraying with water than the comb structure K1 and the double meander structure M1, which both had copper thicknesses of 18 µm (see Table 3). The double spiral S1 underwent a relative change in capacitance (${\bar{C}}_{\mathrm{wet}}/{\bar{C}}_{\mathrm{dry}}$ ,) about 1.5 times higher than the double meander structure M2. The individual values of the capacitance $C_{\mathrm{wet}}$ show relatively large scatter after spraying with water due to the inhomogeneity of the liquid layer, which is also indicated by the standard deviation of the mean value.

**Figure 4:**
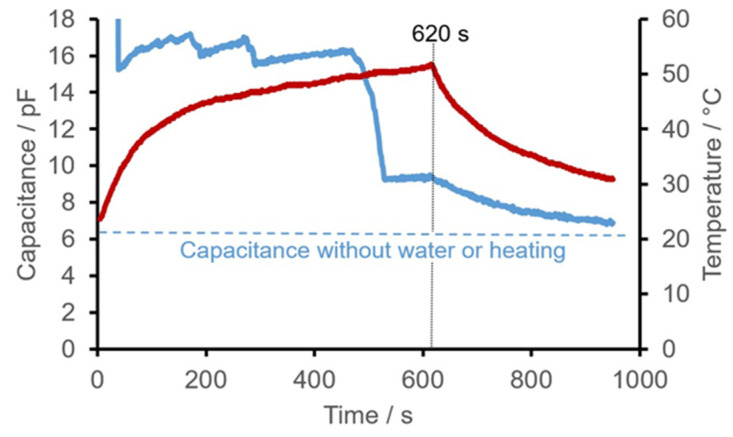
Time courses of electrical capacitance (blue) and temperature (red) of the comb structure K1 after spraying with approximately 1 mL water shown as an example.

Over time, the capacitance of the initially dry sensor structures was almost reached again after spraying with water and subsequent drying by heating after a total of about 1000 s (heating for about 620 s followed by cooling for about 380 s). Periods with very rapid capacitance were observed (see Figure 4).

### 3.2. Rigid Sensor Structures (Ceramic PCBs)

Heating the PCBs from approximately 20 °C to 60 °C on the heating plate caused a relative increase in capacitance of less than 10 %. The capacitance was 38 pF in a dry state ( $C_{\mathrm{dry}}$ , in air), and 310 pF when wet ( $C_{\mathrm{wet}}$ , completely immersed in water).

**Figure 5:**
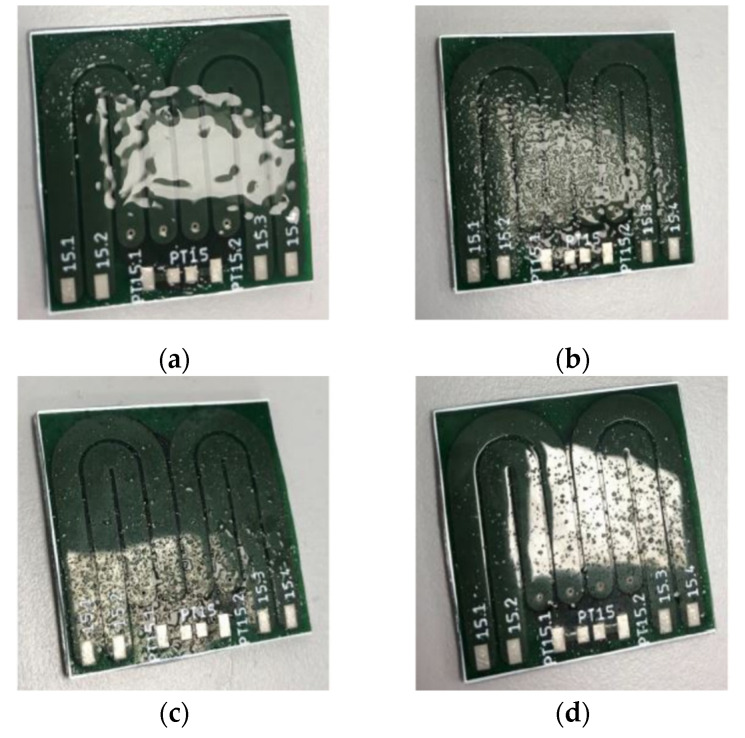
Photos of non-heated printed circuit boards immediately after spraying up hydrogel (1 spray pump, resp.), graded according to increasing spraying distance (5 cm, 10 cm, 15 cm and 20 cm) from top left to bottom right (**a**–**d**).

When hydrogel was sprayed on a printed circuit board that had not been heated up, the following occurred (see Figure 5): At a spraying distance of 5 cm, there was wetting over the entire surface. At a spraying distance of 10 cm, droplets formed on the surface. The greater the spraying distance, the smaller the drops. At all distances, the thickness of the layer of hydrogel appeared to the eye to be inhomogeneous. However, the greater the spraying distance, the less this apparent inhomogeneity. At a distance of 20 cm, it became apparent that one spray burst of hydrogel dried out very quickly at room temperature—probably because of the very small thickness of the layer.

After spraying the inclined sensor structure with pure water (according to Figure 3), the maximum difference in capacitance between the current reading (cur) and the value of the dry PCB (dry) was directly determined. However, repetitions were barely reproducible with regard to the measurements or the course over time, both of which showed large differences.

**Figure 6:**
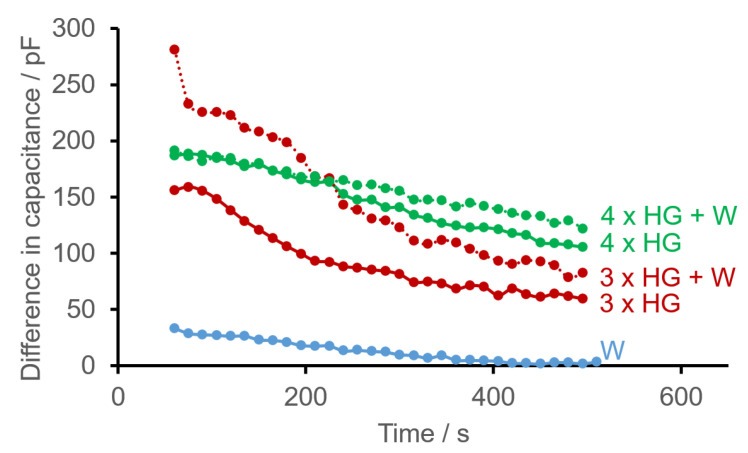
Examples of time courses of differences in capacitance ΔC ( $=C_{\mathrm{cur}}-C_{\mathrm{dry}}$ ) between the current reading (cur) and that of the dry PCB (dry) of a ceramic PCB with the sensor structure double meander M2 after spraying on various combinations of three or four bursts of hydrogel (HG) and water (W). Due to the rather random initial values and stabilization phase of the layer applied, the initial values are not shown for the sake of clarity.

The capacitances decreased and reached the initial values of the dry sensor structure again—probably after the sprayed-on (free) water had completely evaporated—after about 500 s. The difference in capacitance ΔC ( $=C_{\mathrm{cur}}-C_{\mathrm{dry}}$ ) between the current reading (cur) and the value of the dry PCB (dry) was thus zero (again) within the limits of measuring accuracy (Figure 6, blue line). Spraying the inclined sensor structure (according to Figure 3) with hydrogel resulted in a residual capacitance that remained constant from approximately 550 s onwards. The differences in capacitance between the current reading (cur) and the value of the dry PCB (dry) ΔC after 550 s were higher with four spray bursts of hydrogel (Figure 6, green line) than with three (Figure 6, red line). A thicker layer therefore results in a greater difference in capacitance.

Here, too, immediately after spraying, the maximum values of the differences in current capacitance ΔC were reached relatively quickly; the reading did not increase with the number of spray bursts. Deviations between the time courses were also observed, which can be attributed to non-homogeneous application.

Combined spraying of the inclined sensor structure with water and hydrogel (in the experimental set-up shown in Figure 3) revealed that regardless of the number of spray bursts of water and the sequence of sprays with water or hydrogel, the differences in capacitance between the current reading (cur) and the dry PCB (dry) ∆C after 550 s increased with the number of hydrogel spray bursts (see Figure 6). Additional spraying with water evidently does not disturb the residual capacitance of the hydrogel, for the thicker hydrogel layer (4 × HG + W, broken green line) has a higher residual capacitance than the thinner one (3 × HG + W, broken red line) after 550 s. However, both final values are higher than the current values of equal numbers of spray bursts of hydrogel without the additional application of water, presumably indicating that the unbound water had not yet been completely eliminated.

If multiple spray bursts of hydrogel and water are applied to the surface, the maximum values of the current differences in capacitance ∆C are also reached relatively quickly (as when spraying on water or hydrogel). Differences in the time courses were also recorded. A reproducible maximum value with the number of spray bursts at the beginning of the current differences in capacitance $\Delta_{\mathrm{cur}-\mathrm{dry}}C$ was not observed.

As when spraying with water or hydrogel, homogeneous application was not possible when spraying with a combination of hydrogel and water either.

Judging by the estimated thickness of the layer of hydrogel per spray burst at a given distance (see “Hydrogel as a biofilm model”), spraying three times from a distance of 20 cm produced an average thickness of approximately 3 × 4 µm = 12 µm. Accordingly, even hydrogel thicknesses below 20 µm can be detected very well with the setup used (e.g., 3 × HG and 4 × HG in Figure 6).

## 4. Discussion

In order to apply the new method, the sensor structures must be heated. However, the duration of heating should be kept as short as possible to make sure that the biofilm is changed as little as possible. Moreover, the surface temperature during the heating process should be chosen such that the bacteria in the biofilm are not killed while the biofilm continues to grow in the vicinity of the sensor structure. Wherever the method is to be applied, therefore, a temperature profile must be specified that will reliably—but only just—eliminate the unbound water in its entirely.

### 4.1. Flexible Sensor Structures (Polyimide PCBs)

- The increase in capacitance as the temperature rises can only be explained by an increase in the relative permittivity of the insulating material of the PCB and the heating foil. The electrical conductors cannot affect permittivity because the rise in temperature only increases the resistance of the copper.
- A substantial difference in capacitance between dry sensor structures and those wetted with water was to be expected with thicker layers of copper because there are more electric field lines in the area between the conductors where the dielectrics are air or water. Due to the higher absolute differences in capacitance, thicker conductors should be used (Table 1). However, the resulting greater unevenness has a negative impact, since soiling is more likely to stick. Thicker conductors also have lower ohmic resistance, which impedes heating.
- The differences between the average electrical capacitances of the double spiral structure S1 and the double meander structure M2 can be explained by the inhomogeneity of the functional structure (i.e., the structure’s sensitivity). In the double meander structures, the electrical conductors do not directly alternate; instead, the same conductor always lies next to itself in a series of pairs (see Figure 2, left). As a result, every other pair of conductors is at the same electrical potential and does not form an electrical field ‘with itself’. If there is a dielectric in these sections that can be distinguished from air, there is no local change in capacitance there. Due to this effect, only small differences in capacitance can be achieved with meandering structures compared to non-meandering structures with the same area.
- The capacitances of the sensor structures nearly returned to their initial value after being sprayed with water. The fact that the initial value wasn’t quite reached is probably due to the increased permittivity of the film, which is still warmer, and to structural moisture absorption by the polyimide, which is probably not reversible within the time considered. The sharp, rapid changes in capacitance over time can be logically explained by small drops suddenly flowing off. Having formed on the surface during spraying, on reaching a critical size these drops flowed down the inclined PCB and dropped off again (Figure 4).

### 4.2. Rigid Sensor Structures (Ceramic PCBs)

- Non-polar surfaces such as metal or glass tend to be less susceptible to biofouling than polymers [1,14]. However, an essential requirement for a sensor structure is that it should have similar growth dynamics for biofilms to its surroundings. Therefore, a sensor structure with a glass surface is probably far more suitable than a sensor with a polyimide surface for infrastructure systems with predominantly metallic surfaces.
- The much smaller increase in capacitance with temperature in rigid PCBs compared to flexible PCBs can be well explained by the temperature dependence of the relative permittivity of the different materials. The relative permittivity of polyimide ( $\varepsilon_{r,PIm}\approx 3.4$ ) [25] shows a much stronger temperature dependence than that of ceramics and glass (Alumina Ceramic: $\varepsilon_{r,AlC}=9$ [26].
- With the ceramic PCBs, greater differences in capacitance between the measurements in air (dry) and in water were to be expected, because, compared to the polyimide PCBs, they were not just wetted with water during measurement but were completely immersed in water. As a result, the maximum capacitance change with the structure used was approximately 800%, roughly corresponding to the theoretical maximum possible change.
- The non-uniform layer thickness of the hydrogel (Figure 5), which was found in experiments to be dependent on the spraying distance, is not thought to present a problem because in order to detect a biofilm, only a reliable detection threshold needs to be determined above which a biofilm can be identified, and no quantification (such as the thickness or the water content of the biofilm, etc.) is required.
- If only water is applied to a sensor structure, the maximum difference in capacitance is recorded immediately afterwards, as is to be expected. However, when measured again, large differences in the temporal course occur despite the identical number of sprays, which is almost certainly due to drops of water on the sensor surface randomly converging and then draining off. If water is sprayed more than 10 times, drops form immediately and then converge on the underside of the PCB due to its inclined position. However, all the measurements carried out indicated a decrease in capacitance down to the initial value, which was reached at the end of the observation period (apart from scatter).
- If hydrogel is sprayed onto the surface of a sensor structure multiple times, the maximum values of the current differences in capacitance ΔC are also reached relatively quickly. Deviations also exist in the time courses. Again, a maximum value correlating with the number of spray bursts cannot be observed initially because—like water—homogeneous application with hydrogel was not possible either. However, it is clearly apparent (Figure 6) that as the thickness of the layer of hydrogel increases (due to more spray bursts), there is a higher difference in capacitance ΔC on average after 550 s.
- If water and hydrogel are applied to the surface, the initial values differ even more clearly, albeit again without a recognizable pattern, which is probably also due to inhomogeneous application.
- By estimating the layer thickness of hydrogel applied per spray burst at a given distance, even layer thicknesses below 20 µm can be detected very well with the setup used.

### 4.3. Conclusions

A new method for the detection of microbial biofilms is described. Like other known detection methods, its range of application is limited. Damp environments that are not permanently underwater such as exhaust systems containing condensation moisture (e.g., in air conditioning systems, infrastructure in the food processing industry) are particularly suitable areas of application. Furthermore, capacitive sensor structures could be installed in inspection shafts that can be drained at regular intervals (perhaps automatically) so that measurements can be taken, for instance in infrastructure systems such as dental water systems or the water supply network. Additionally, they could be used in dishwashers, washing machines and fully automatic coffee machines to detect the formation of biofilms in such appliances in good time.

One major advantage of this process is clearly its robustness and simplicity. Due to the largely chemically inert hard glass surface, it is likely to be very resistant to wear caused by mechanical and chemical environmental conditions. This should be particularly emphasized in the light of biofilm-induced corrosion (biofouling) [25]. A sensor like this is far less likely to be affected by mechanical or chemical wear, unlike for example electrodes based on a biochemical reaction (e.g., an amperometric glucose electrode with glucose oxidase for measurements in tissue) [27].

So far, however, the sensor structure has only been tested on a technical model. Measurements with real bacterial cultures to validate the model are planned. How temperature affects bacteria during the heating process also needs to be ascertained. The suitability of the sensor surface as a biofilm substrate compared to the surrounding surface is still unknown, too. Further investigations are to be carried out to improve the method (e.g., an environment-specific surface coating), to identify practical areas of application, and to assess its suitability for detecting real biofilms.

## Figures and Tables

**Table 1:** The table shows the geometries of the conductor layers of the flexible PCBs (height, width and spacing of copper).

Structure	Thickness of Copper	Width of Copper	Spacing
S1	28 µm	1.6 mm	0.4 mm
K1	18 µm	1.6 mm	0.4 mm
M1	18 µm	1.6 mm	0.6 mm
M2	28 µm	1.6 mm	0.6 mm

**Table 2:** The table shows the applied mean layer thickness of hydrogel per spray (measured in microns) applied to the sensor-structure with the method described above.

Spray Distance in cm	5	10	15	20
Mean layer thickness (µm; SD: ±0.8 µm)	44	18	8	4

**Table 3:** Mean capacitances (( $\bar{C}=\left(C_{\max}-C_{\min}\right)/2$ ), the difference in electrical capacitances in moist (wet) and dry (dry) states ΔC (= ${\bar{C}}_{\mathrm{wet}}-{\;\bar{C}}_{\mathrm{dry}}$ ), and the relative capacitance changes ( ${\bar{C}}_{\mathrm{wet}}/{\bar{C}}_{\mathrm{dry}}$ ) of the four sensor structures (three to five measurements each). The structures were dry or moistened by spraying with water.

Structure	${\bar{C}}_{dry}/\mathrm{pF}$	${\bar{C}}_{wet}/\mathrm{pF}$	ΔC/pF	${\bar{C}}_{wet}/{\bar{C}}_{dry}$
S1	29 ± 1	90 ± 18	61 ± 19	3.1 ± 0.7
K1	6 ± 1	17 ± 3	11 ± 4	3.0 ± 1.0
M1	8 ± 1	34 ± 13	26 ± 14	4.5 ± 2.3
M2	30 ± 5	58 ± 12	28 ± 17	2.1 ± 0.7

## Data Availability

Data sharing not applicable, all relevant data are contained in the article.
